# Genistein contributes to cell cycle progression and regulates oxidative stress in primary culture of osteoblasts along with osteoclasts attenuation

**DOI:** 10.1186/s12906-020-03065-5

**Published:** 2020-09-11

**Authors:** Sahabjada Siddiqui, Abbas Ali Mahdi, Md Arshad

**Affiliations:** 1 Department of Biotechnology, Era’s Lucknow Medical College & Hospital, Era University, Lucknow, 226003 India; 2 Department of Zoology, University of Lucknow, Lucknow, 226007 India; 3 Department of Biochemistry, King George’s Medical University, Lucknow, 226003 India; 4 Department of Zoology, Aligarh Muslim University, Aligarh, 202002 India

**Keywords:** Cell cycle, Isoflavone, Oxidative stress, Primary bone cells

## Abstract

**Background:**

The present study was designed to examine the role of isoflavone genistein (GS) on bone formation, regulating oxidative stress and cell cycle in primary osteoblasts, as well as attenuation of osteoclast formation.

**Methods:**

Primary calvaria osteoblasts were isolated from 2 to 3 days old neonatal rat pups (*n* = 6–8) of Sprague Dawley rats. Osteoblasts were incubated with varying concentrations of GS and different assays *viz.* cell proliferation, differentiation, calcium deposition, cell cycle progression, antioxidant ability, and osteogenic gene expression were performed. Tartrate-resistant acid phosphatase (TRAP) staining and immunolocalization of cathepsin K protein were assessed in bone marrow-derived osteoclasts.

**Results:**

Results revealed that GS markedly induced cell growth and osteoblast differentiation depending upon dose. The fluorescent dye DCFH-DA staining data proved the antioxidant ability of GS, which reduced the H_2_O_2_- induced intracellular oxidative stress in osteoblasts. Quantitative real-time PCR analysis revealed that GS treatment upregulated the expression of osteoblastic genes of Runt-related transcription factor 2 (Runx2), bone morphogenetic proteins 2 (BMP2), and osteocalcin. Immunolocalization of BMP2 also indicated the osteogenic efficacy of GS. Furthermore, TRAP staining and cathepsin K expression depicted that GS inhibited multinucleated osteoclasts formation.

**Conclusions:**

In conclusion, GS isoflavone might impart protective effects against oxidative stress-induced bone loss and thus, could maintain skeletal growth.

## Background

The balance between bone formation, bone resorption, proliferation, and apoptosis helps to maintain bone mass nearly constant [1]. An imbalance between bone resorption and bone formation may cause metabolic disorders or bone pathological conditions of the adult skeleton and lead to low bone mass, osteoporosis, and increased bone fracture risk [2]. Based on available data and clinical experiences, about 200 million women worldwide are osteoporotic and report approximately 8.9 million fractures annually [3]. This is equivalent to an osteoporotic fracture every 3 s in the woman [4]. The most common risk factors for osteoporotic fracture are deteriorating bone mineral density along with advanced age [5]. Therefore, diet strategies, including functional foods and nutraceuticals, can be valuable assets for the peak bone mass of an individual and can support bone health during later life stages. Thus, with a rise in the life expectancy of people, the need for prevention and bone preservation measurements is greater than ever.

For osteoporosis treatments, most pharmaceuticals concentrate on inhibition of osteoclastogenesis to retard bone resorption, while preventive strategies are most important. Previous studies have shown that isoflavones and flavonoids increase bone mass density and prevent bone loss in-vivo and in-vitro [6–8]. Thus, flavonoids and isoflavones have the potential to maintain and improve the bone mass of human subjects. Flavonoids and isoflavones are secondary metabolites found in various plant parts such as fruits, vegetables, grains, flowers, roots, barks and stems [9]. Genistein (4′,5,7-trihydroxyisoflavone, GS) structurally belongs to the group of isoflavones which have potential to be developed into antiosteoporotic and anticancer drugs. GS is present in various food items primarily in soybeans and soy products, which is capable of interacting with various hormones like estrogens [10, 11].

Several studies have reported the osteogenic activity of various herbal compounds on the primary culture of rodent bone cells which is an easy available model to study the fundamental processes involved in bone formation and relevant for clinical studies. Icariin, a prenylated flavonol glycoside, has been reported to have potential for bone cell formation using rat calvarial osteoblast culture [12]. Likewise, a Chinese medicinal plant, *Alpinia officinarum*Hance., has shown osteogenic activity in both ovariectomized Sprague Dawley (SD) rats as well as in primary osteoblasts and has a protective role against the lethal effects of intracellular ROS generation [13]. Mice calvaria osteoblasts have been tested in vitro for osteoblast mineralization using water extract of *Rumex crispus* root [14]. In addition, ethanolic extract of *Punica granatum* and methanolic extracts of mistletoe leaves and their isolated compounds have shown osteogenic potential in primary rat calvaria culture [15, 16]. Genistein has also been reported to prevent trabecular bone loss of proximal tibia in a male rat model of osteoporosis [17] and in combination with silicon, it also protects ovariectomized bone loss by upregulating OPG/RANKL ratio [18]. In addition, previous studies have reported that genistein inhibits lipopolysaccharide-stimulated inflammatory and oxidative stress response as well as RANKL-stimulated osteoclast differentiation using RAW 264.7 macrophage cell lines [19, 20]. However, further studies are needed to justify the role of GS in regulation of cell cycle and oxidative stress in primary culture of bone cells along with osteoclasts attenuation in bone marrow progenitor cells.

In this study, cultured bone cells were exposed to different concentrations of GS and the effect of GS on cell cycle progression and oxidative stress along with osteogenic gene expression were analyzed in primary osteoblasts. Inhibition of osteoclasts differentiation by GS was analyzed in bone marrow-derived osteoclast cells. Our findings indicated that GS has beneficial effects on bone regeneration and preservation.

## Methods

### Reagents and chemicals

Alpha- minimum essential medium (a-MEM), foetal bovine serum (FBS), MTT powder, p-nitrophenyl phosphate (pNPP), naphthol AS-MX phosphate, fast blue BB salt, ascorbic acid and Alizarin Red S dye were purchased from Himedia, India. Propidium iodide (PI), β-glycerophosphate, 2,7-dichlorodihydrofluorescein diacetate (DCFH-DA) and 17β-estradiol (E2) were purchased from Sigma-Aldrich, USA. RNAiso Plus reagent was procured from Takara, India. Revert Aid First Strand cDNA Synthesis Kit was procured from Thermo Scientific, USA and LightCycler® 480 SYBR Green I kit was purchased from Roche, Germany. Anti-BMP2 antibody and anti-cathepsin K antibody were procured from Molecular Probes, USA. All other chemicals were of analytical grade.

### Culture and maintenance of osteoblasts

For isolation and culrture of bone cells, SD rats were obtained from Central Drug Research Institute, Lucknow, India and the protocols employed were ethically approved by Institutional Animal Ethics Committee of Azad Institute of Pharmacy and Research, Lucknow, India (Ref No. AIPR/2013–14/1398).

Osteoblasts were isolated from the calvaria of rat pups by sequential digestion as per established protocols [16]. Briefly, 2–3 days old neonatal rat pups in number 6–8 were cervical dislocated by applying firm pressure at the base of skull, sharply pinching and twisting between thumb and forefinger and at the same time, pulling backward on the tail. Calvaria was excised with the help of scissors, scalpels, and forceps. Isolated calvaria was subjected to five successive digestions in a solution containing 0.1% collagenase II and dispase II enzymes at 120 rpm and 37 °C for 10–15 min in a shaking water bath. Supernatants from second to fifth digestions were collected in a 15 ml tube containing 800 μl FBS and then centrifuged. Pellets were resuspended in complete α-MEM media and cultured in T^− 25^ cm^2^ flasks at 37 °C with 5% CO_2_ in a CO_2_ incubator (Excella ECO-170, New Brunswick).

### Isolation and culture of bone marrow progenitor cells

For osteoclast culture, bone marrow progenitor cells from femur and tibia bones of rodents were isolated as per described protocol with minor modifications [21]. Briefly, 4–6 days neonatal rat pups were cervical dislocated as mentioned previously. Long bones of neonatal rats were isolated and cleared the soft tissues using sterile surgical blades. Bones were washed with ice-cold PBS, cut lengthwise and then chopped finely using a scalpel blade in a petri-dish containing α-MEM without FBS. The cell suspensions containing bone marrow were collected in a tube and centrifuged at 1500 rpm for 5 min. Settled cells were cultured in α-MEM containing heat-inactivated FBS and M-CSF (30 ng/ml) at 37 °C and 5% CO_2_. After 24 h incubation, overlaying medium was discarded and settled cells were pooled in a tube by gentle washing with sterile PBS. The cell suspension was centrifuged at 1500 rpm for 5 min and pellet was re-cultured in osteoclast differentiating media (osteoclast media containing 10% FBS, antibiotics solution, 10^− 8^ M vitamin D_3_ and 10^− 6^ M dexamethasone + M-CSF 30 ng/ml + RANKL 50 ng/ml).

### Osteoclastogenesis and treatment

Fresh osteoclast differentiating media containing desired concentrations (1, 5 and 10 μM) of GS was used to replace old media of cultured osteoclasts in a 24-well plate at every 3 days. After 9 days of treatment, cells were used for TRAP staining, Cathepsin K gene expression analysis and their immunofluorescence staining. Multinucleated cells containing ≥3 nuclei were scored as TRAP-positive osteoclast cells.

### Cell proliferation analysis

The proliferative effect of GS was determined by MTT assay as per protocol [22]. Briefly, cultured osteoblasts at a density of 2 × 10^3^ cells/well were seeded in 96-well plate and incubated with GS solution prepared in culture media at concentrations 1, 5 and 10 μM in triplicate for 48 h. The 17β-estradiol (E2) at 1 nM was considered as a positive control because it can produce maximum induction in osteoblasts as per organisation for economic co-operation and development (OECD) guidelines. MTT reagent was added and the colored product was read at 540 nm using a microplate reader (BIORAD-680, USA). Cells growth was analyzed by using the equation:

$$ \%\mathrm{Cells}\kern0.34em \mathrm{Growth}=\frac{\mathrm{Absobance}\kern0.17em \mathrm{of}\kern0.17em \mathrm{treated}\kern0.17em \mathrm{cells}}{\mathrm{Absobance}\kern0.17em \mathrm{of}\kern0.17em \mathrm{control}\kern0.17em \mathrm{cells}}\times 100 $$

### ALP activity of osteoblasts

ALP activity of osteoblasts was evaluated using the following protocol [15]. Briefly, osteoblasts at density 2 × 10^3^cells/well were seeded in a 96-well plate using osteoblast differentiation medium containing complete α-MEM supplemented with 10 mM β-glycerophosphate and 50 μg/ml ascorbic acid. After overnight, cells were incubated with GS at concentrations 1, 5 and 10 μM for 48 h. Treated and untreated cells were fixed in 4% paraformaldehyde and then stained with ALP reagent (naphthol AS­MX phosphate at 0.1 mg/ml, N, N- dimethylformamide at 0.5%, MgCl_2_ at 2 mM, and fast blue BB salt at 0.6 mg/ml in Tris-HCl solution of 0.1 mM and pH 8.5) for 30 min. Images were captured under an inverted phase-contrast microscope (Nikon ECLIPSE Ti-S, Japan). To quantify the stain, the plate was placed at -70 °C for 20 min, and then freeze fractured at 37 °C. The chilled pNPP substrate solution was added and incubated further at 37 °C for color development. Absorbance was read at 405 nM with the help of ELISA reader.

### Calcium deposition assay

Calcium content in osteoblasts was measured using Alizarin Red S dye after 3 weeks of treatment periods following a previous protocol [15]. Approximately, osteoblasts at a density 2 × 10^4^ cells/well were cultured in osteoblast differentiation medium containing 10^− 7^ M dexamethasone in a 12-well culture plate. Cells were incubated with and without GS at desired concentrations for 21 days at 37 °C in a CO_2_ incubator. The medium was replaced every 48 h with fresh treatment for 21 days. Cells were then fixed with 4% paraformaldehyde for 15 min and then rinsed twice with PBS followed by Alizarin Red-S staining (40 mM, pH 4.5) for 30 min. Stained calcified nodules were photographed using inverted phase-contrast microscopy. Alizarin stain was extracted with 100 mM cetylpyridinium chloride solution and released calcium-bound alizarin red solution was used for colorimetric quantification at 570 nm using a microplate reader (BIORAD-680, USA).

### DNA content analysis in S-phase of the cell cycle

DNA content analysis in S-phase of cells population by flow cytometry is the method of cell proliferation. For this study, primary osteoblast cells were seeded at density 1 × 10^6^ cells/ml in 6-well culture plates and then treated with GS at desired concentrations for 48 h. Flow cytometry study was carried out to analyze the cellular DNA content using standard protocol [15]. The fluorescence intensity of stained nuclei was measured with the help of flow cytometer (BD FACS Calibur, Becton Dickinson, USA) and data were analyzed.

### Measurement of the antioxidant ability of GS

Hydrogen peroxide (H_2_O_2_) solvent was used as an exogenous ROS source inducing oxidative stress and free radicals in osteoblasts. The osteoblast cells were incubated with various concentrations of H_2_O_2_ (10–100 μM) for 24 h and the IC_50_ value was evaluated as described previously [22]. Further, to investigate the antioxidant ability of GS on osteoblasts, the confluent cells were pre-treated with 1, 5 and 10 μM concentrations of GS for 24 h and then treated with or without Sub-IC_50_ value (40 μM) of H_2_O_2_ for 24 h. After incubation period, cells were stained with DCFH-DA dye for 20 min and imaged from 5 to 7 random fields of each sample under an inverted fluorescence microscope (Nikon ECLIPSE Ti-S, Japan). For ROS quantification, osteoblasts were incubated with DCFH-DA dye in PBS and the fluorescence was quantified at excitation wavelength 485 nm and emission wavelength 528 nm by a multiwell microplate reader (Synergy H1 Hybrid Multi-Mode Microplate Reader, BioTek, USA).

### Real-time quantitative PCR (qPCR) analysis

Both osteoblasts and osteoclast cells were treated with different concentrations of GS and gene expression analysis was performed following a previous protocol [23]. Briefly, total RNA was isolated from cultured osteoblasts using RNAiso Plus reagent (Takara, India) following the manufacturer’s instructions. A 1 μg of isolated RNA was used in 20 μl reaction volume and cDNA was synthesized using Revert Aid First Strand cDNA Synthesis Kit (Thermo Scientific, USA). Concentrations of cDNA were measured by spectrophotometer and 12.5 ng/μl cDNA was used for qPCR reaction. For absolute quantification of both osteogenic and osteoclastic gene expression, real-time PCR analysis was performed in a light cycler PCR system (Light Cycler 480, Roche, Germany) using LightCycler® 480 SYBR Green I kit (Roche, Germany). For absolute quantification, a standard curve was prepared from treated and untreated cells of known template concentration and the concentration of the unknown sample was determined by interpolation of its PCR signal (cycle threshold) into the standard curve. The sequence of primer pairs is listed in Table S1.

### Immunofluorescence staining of BMP2 protein

Immunofluorescence staining was performed as per the previous protocol [23]. Treated and untreated cells were fixed in 4% paraformaldehyde and permeabilized with permeabilizing buffer. Cells were washed with PBS and then blocked with blocking buffer at RT for 1 h to avoid non-specific binding. Cells were then incubated with a rat anti-BMP2 antibody (1:1000 dilution, Abcam, Cambridge, UK) containing 0.5% BSA at 4 °C for overnight. After washing with PBS, cells were incubated with fluorescent Alexa Fluor-488 conjugated anti-rat BMP2 antibody (1:1000 dilution, Molecular Probes, USA) for 1 h at RT. Cells were washed with PBS and then photographed under a fluorescent microscope (Nikon ECLIPSE Ti-S, Japan). BMP2-stained cells were counted in five randomly selected fields from three wells of each treatment and analyzed with Image-Pro Plus 6.1 software (Media Cybernetics, Rockville, MD, USA).

### TRAP staining assay

TRAP staining was performed as per the described protocol [24]. Briefly, cultured cells in a 24-well plate were fixed with fresh 4% paraformaldehyde for 10 min. Subsequently, 0.5 ml of staining reagent (5–10 mg Naphthol AS-MX phosphate in 0.5 ml dimethylformamide + 50 ml acetate-tartrate buffer + 20 mg fast red violet LB salt) was added in each well and incubated at RT for 5–10 min. Cells were washed twice with distilled water and photographs were taken by an inverted phase-contrast microscope.

### Immunofluorescence staining of Cathepsin K

Cathepsin K activity was assayed qualitatively by cathepsin K immunofluorescence staining following protocol with little modification [25]. Cells were fixed in 4% paraformaldehyde, permeabilized with permeabilization buffer, and then blocked with blocking buffer at RT for 1 h. Next, cells were incubated with rat anti-cathepsin K antibody (1:1000 dilution, Abcam, Cambridge, UK) containing 0.5% BSA at 4 °C for overnight. After the incubation period, cells were then washed with PBS and incubated with fluorescent Alexa Fluor-488 conjugated anti-rat cathepsin K antibody (1:1000 dilution in PBS) (Molecular Probes, USA) for 1 h at RT. Stained cells were photographed using a fluorescent microscope (Nikon ECLIPSE Ti-S, Japan). Cathepsin K- stained cells were counted in five randomly selected fields from three wells of each treatment and analyzed using Image-Pro Plus 6.1 software.

### Statistical analysis

All triplicates data were represented as means ± SEM. One-way ANOVA and Dunnett’s multiple comparison tests were implemented for statistical significance. Student t-test was also applied as per experimental need using Microsoft Excel and Roche software. A probability value of P<0.05 was considered statistically significant.

## Results

### Effect of GS on osteoblasts growth

**Fig. 1:**
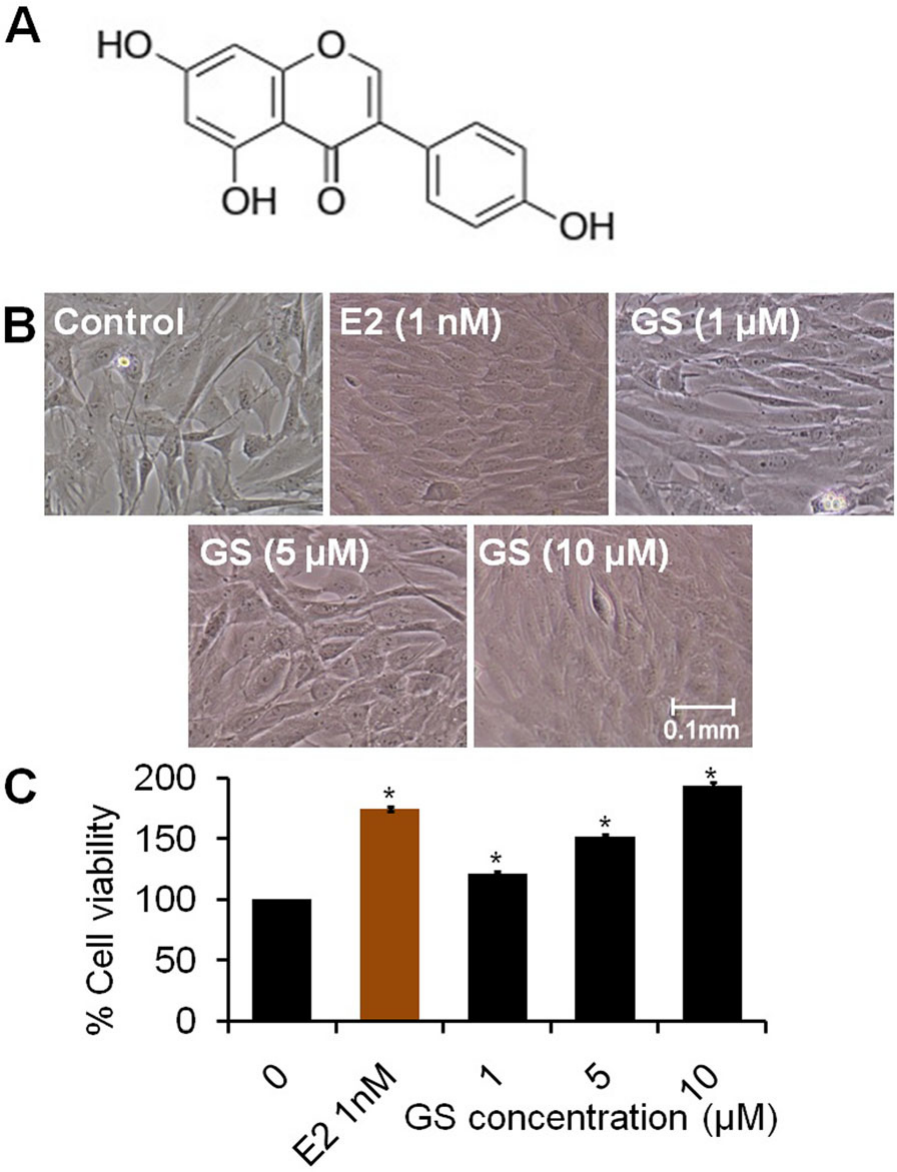
Microscopic observation and percent cell proliferation of primary osteoblasts. (**a**) Chemical structure of Genistein (**b**) Morphology of osteoblasts under inverted phase-contrast microscope treated with different concentrations 1, 5 and 10 μM of GS at 48 h. Scale bars = 0.1 mm (**c**) Percent cell proliferation of osteoblasts treated with different concentrations of GS. Cells treated with 1 nM of E2 was used as a positive control. Values were obtained from three independent experiments and expressed as mean ± SEM. ^*^P < 0.05 as compared with control

The elongated and fusiform appearance of osteoblasts was observed under an inverted phase-contrast microscope. Compact and dense layering of cells at high doses revealed growth of osteoblast cells (Fig. 1b). Figure 1a represents the chemical structure of genistein. Data of MTT assay revealed that 1, 5 and 10 μM concentrations of GS resulted in approximately 121.66, 151.50, and 193.23% growth as compared to control. Exposure of cells with 1 nM of E2 (positive control) increased the growth of cells to 174.84% as compared to control (Fig. 1c). MTT data was consistent with the morphological data of osteoblasts.

### Effect of GS on ALP activity

**Fig. 2:**
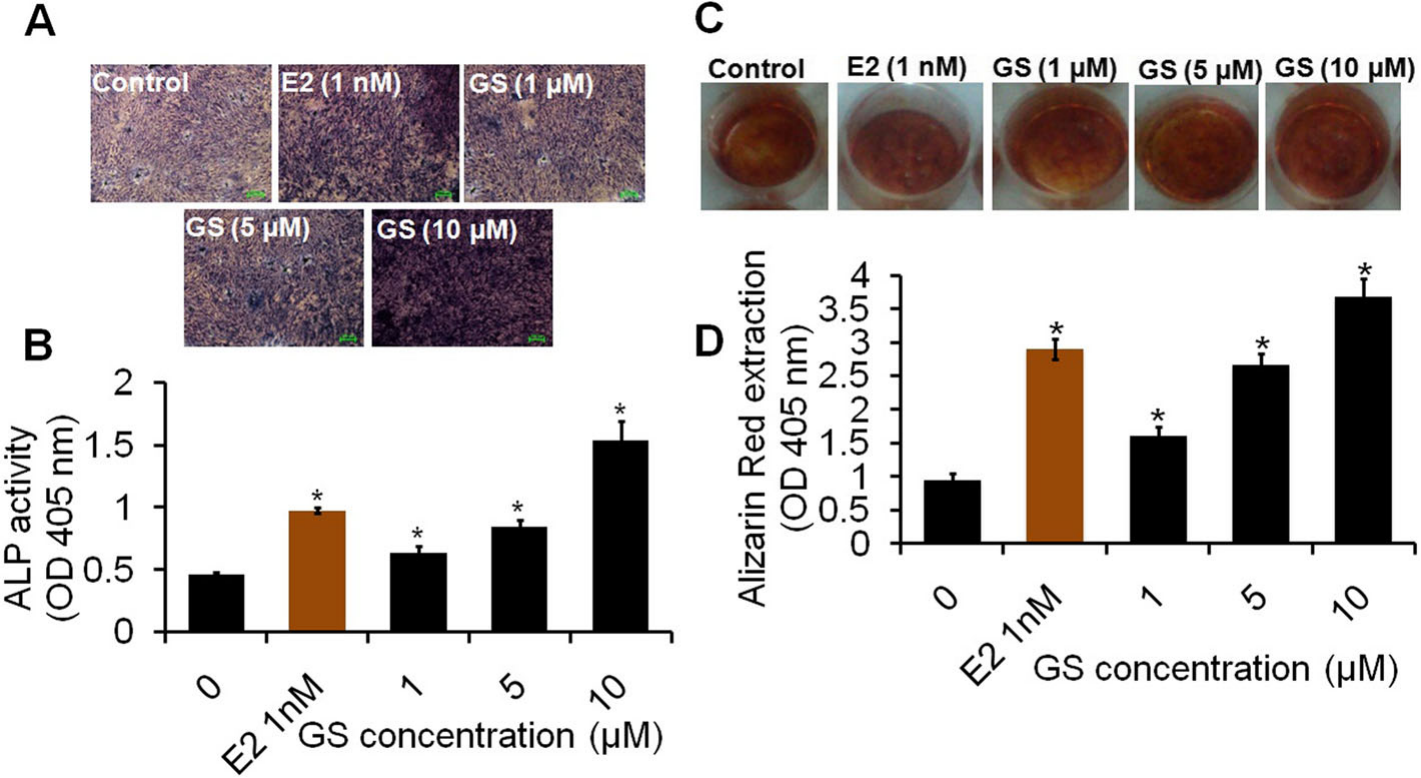
Effect of GS on osteoblasts differentiation and calcification (**a**) Photomicrographs show ALP stain in osteoblasts treated with increasing concentrations 1, 5 and 10 μM of GS at 48 h (**b**) Quantitative data of ALP level are presented in the form of ALP activity relative to control (**c**) Photomicrographs represent the increased formation of mineralized nodules of osteoblasts treated with increasing concentrations 1, 5 and 10 μM of GS at 21 days. Scale bars = 0.1 mm (**d**) Quantitative data of Alizarin Red-S extraction are expressed in the form of percent calcification. Data are represented as mean ± SEM of three independent experiments. ^*^P < 0.05 as compared with control

ALP is an early differentiation marker of osteoblasts and the amplified expression of ALP is highly correlated with the progressive differentiation of osteoblasts. Data of ALP stain showed that GS increased the ALP stain upon increasing the concentrations of GS (Fig. 2a). The quantitative data of ALP is represented in the form of absorbance at different concentrations of GS treatment. As observed from the results (Fig. 2b), GS induced cell differentiation significantly (*P <* 0.05) depending upon dose. Cells exposed with 1 nM of E2 also increased the ALP activity as compared to control.

### GS induces mineralization of osteoblasts

As revealed by results, osteoblasts displayed monolayer fibroblastic appearance in the proliferation period in control cells. Cells treated with various concentrations of GS appeared to form mosaic-like multiple layers at 1, 5 and 10 μM of doses (Fig. 2c). After 21 days, the quantitative calcification data of osteoblasts at different concentrations of GS is shown in Fig. 2d. As observed from the data, GS increased the calcified nodules formation in cultured osteoblasts, which exhibits significant osteogenic effect (*P <* 0.05) as a function of dose.

### DNA content analysis

**Fig. 3:**
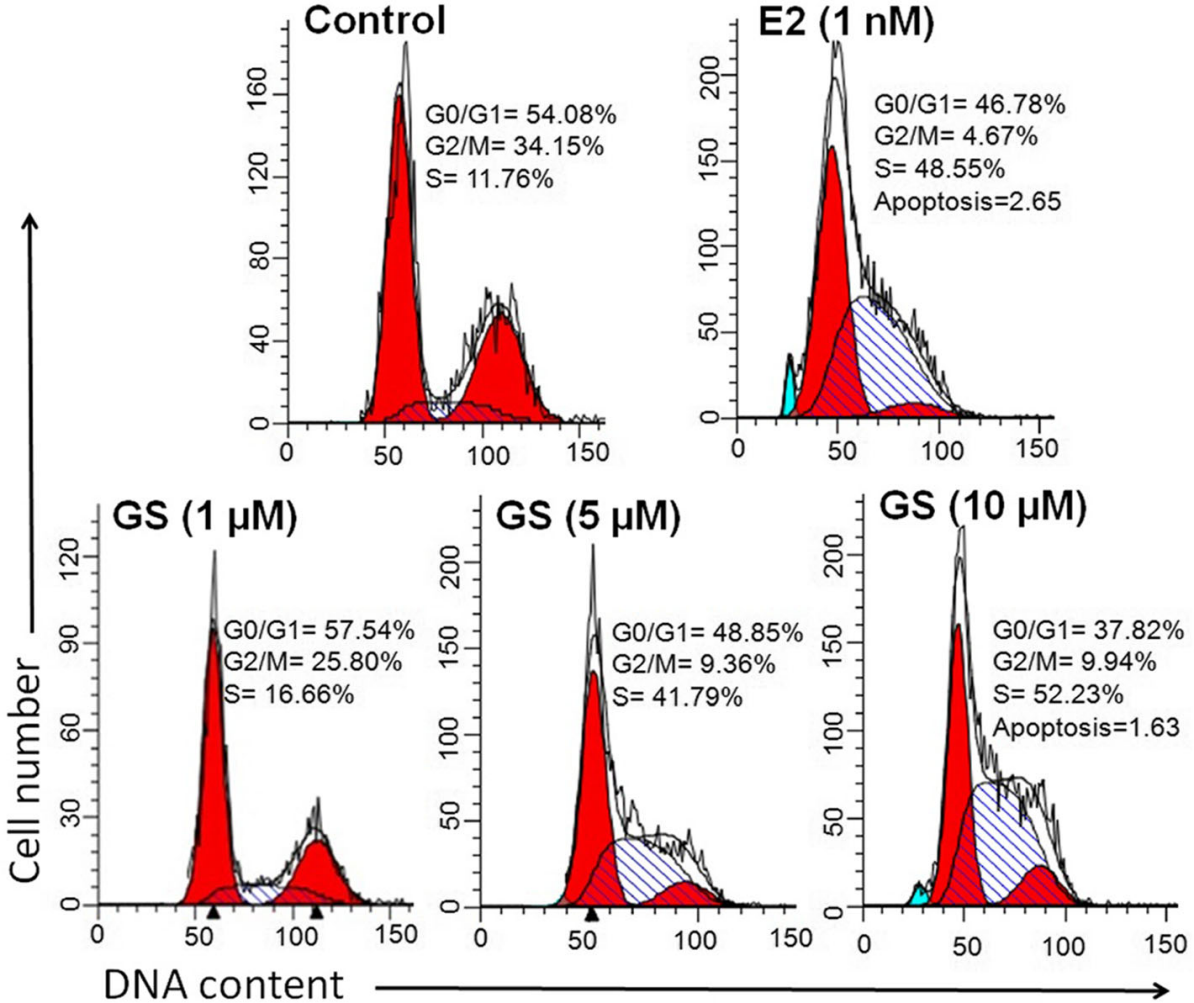
Analysis of cell cycle progression by flow cytometry. Pictorial graph showing the proportion of cells in different phases of cell cycle treated with 1, 5, and 10 μM of GS at 48 h. Cells treated with 1 nM of E2 was used as a positive control

As observed from the result, the normal distribution of different phases of the cell cycle was found in the control group. As compared to control, % DNA content in S phase of cell cycle was sharply increased from 11.76 to 16.66% at 1 μM of GS treatment. A notable increase in the percent DNA content of S phase was observed by 41.79 and 52.23% at 5 and 10 μM of GS treatment (Fig. 3). Exposure of 1 nM E2 compound to osteoblasts increased the % DNA content in S phase by 48.55%.

### GS reduced the oxidative stress of osteoblasts

**Fig. 4:**
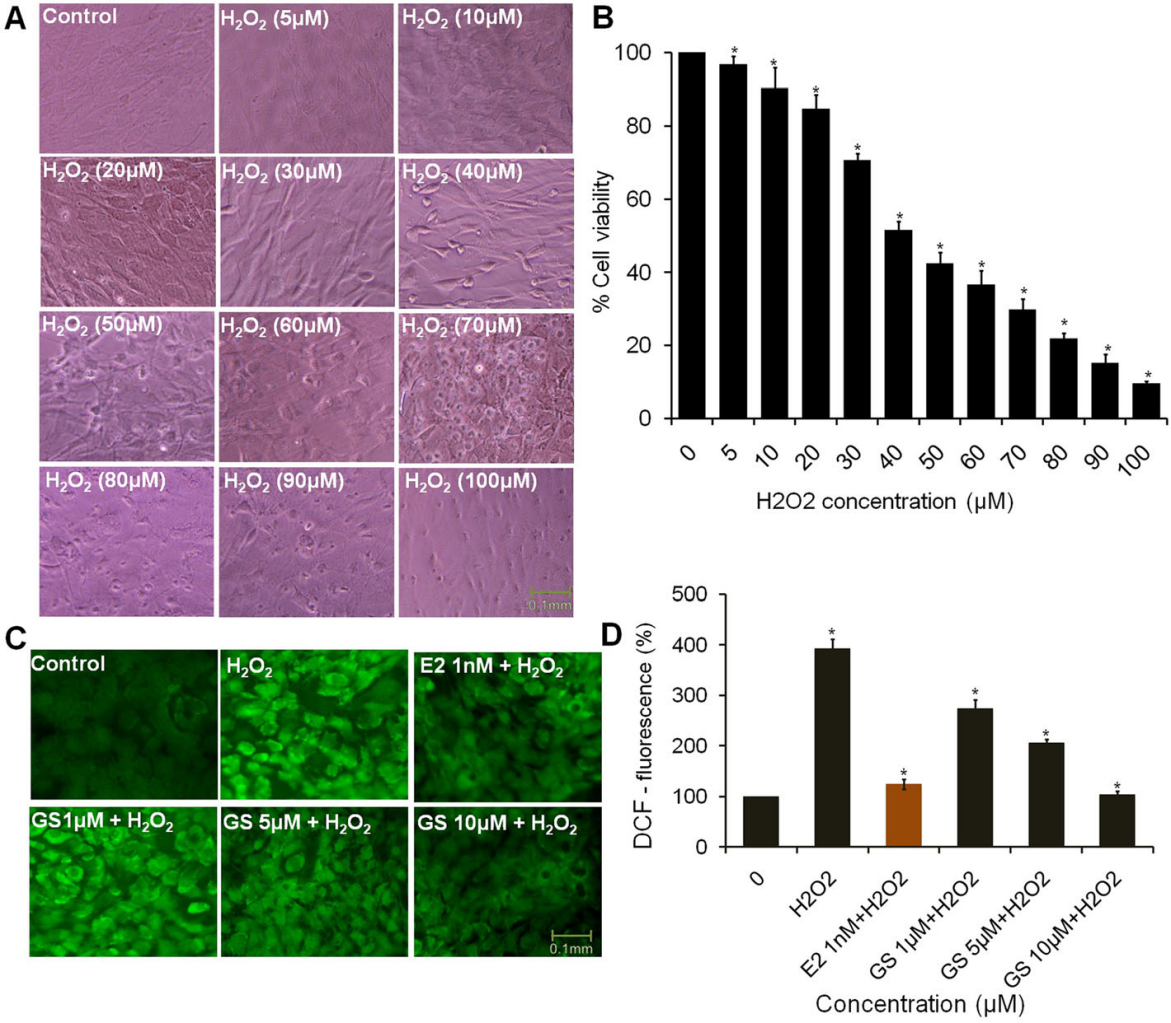
Protective effect of GS on H_2_O_2_-induced cytotoxicity and intracellular oxidative status in osteoblasts. **a** Concentration-dependent effects of H_2_O_2_ exposure on the morphology of osteoblasts after 24 h (**b**) Concentration-dependent effects of H_2_O_2_ exposure on viability of osteoblasts (**c**) Representative photomicrographs show intracellular ROS generation stained with DCFH-DA dye, Scale bar = 100 μm (**d**) Quantitative measurement of ROS level of osteoblasts was expressed as the percentage of DCF-fluorescence relative to controls. Values were obtained from three independent experiments and expressed as mean ± SEM. ^*^P < 0.05 compared with control

To evaluate the antioxidant effect of GS, H_2_O_2_ was employed as an exogenous ROS source to induce oxidative stress in osteoblasts. H_2_O_2_ treatment reduced the number of cells considerably as compared to untreated cells (Fig. 4a). MTT data showed that H_2_O_2_ decreased the percent viability of osteoblasts depending upon dose (Fig. 4b). The cell viability data of osteoblasts was consistent with decreasing number of treated osteoblasts. The IC_50_ value of H_2_O_2_ was found 45.8 μM against osteoblasts. Therefore, a sub-IC_50_ value of H_2_O_2_ i.e. 40 μM was used to induce osteoblasts oxidative stress in later experiments. Pre-treated osteoblasts with GS at 1, 5 and 10 μM concentrations for 24 h followed by 40 μM H_2_O_2_ incubation for another 24 h, GS significantly attenuated the H_2_O_2_-induced injury depending upon dose. These results suggests that GS may suppress H_2_O_2_-induced cell death. As shown in Fig. 4c, after 24 h of H_2_O_2_ incubation, the DCFHDA fluorescence intensity of osteoblasts was increased significantly as compared to control. This result suggests that enhanced intracellular ROS level of osteoblasts may result into damage of their cellular function. On the other hand, ROS generation was significantly inhibited by GS at its each concentrations of treatment. Reference control E2 at 1 nM concentration reduced the ROS level as compared to control.

### qPCR analysis of osteogenic genes expression

**Fig. 5:**
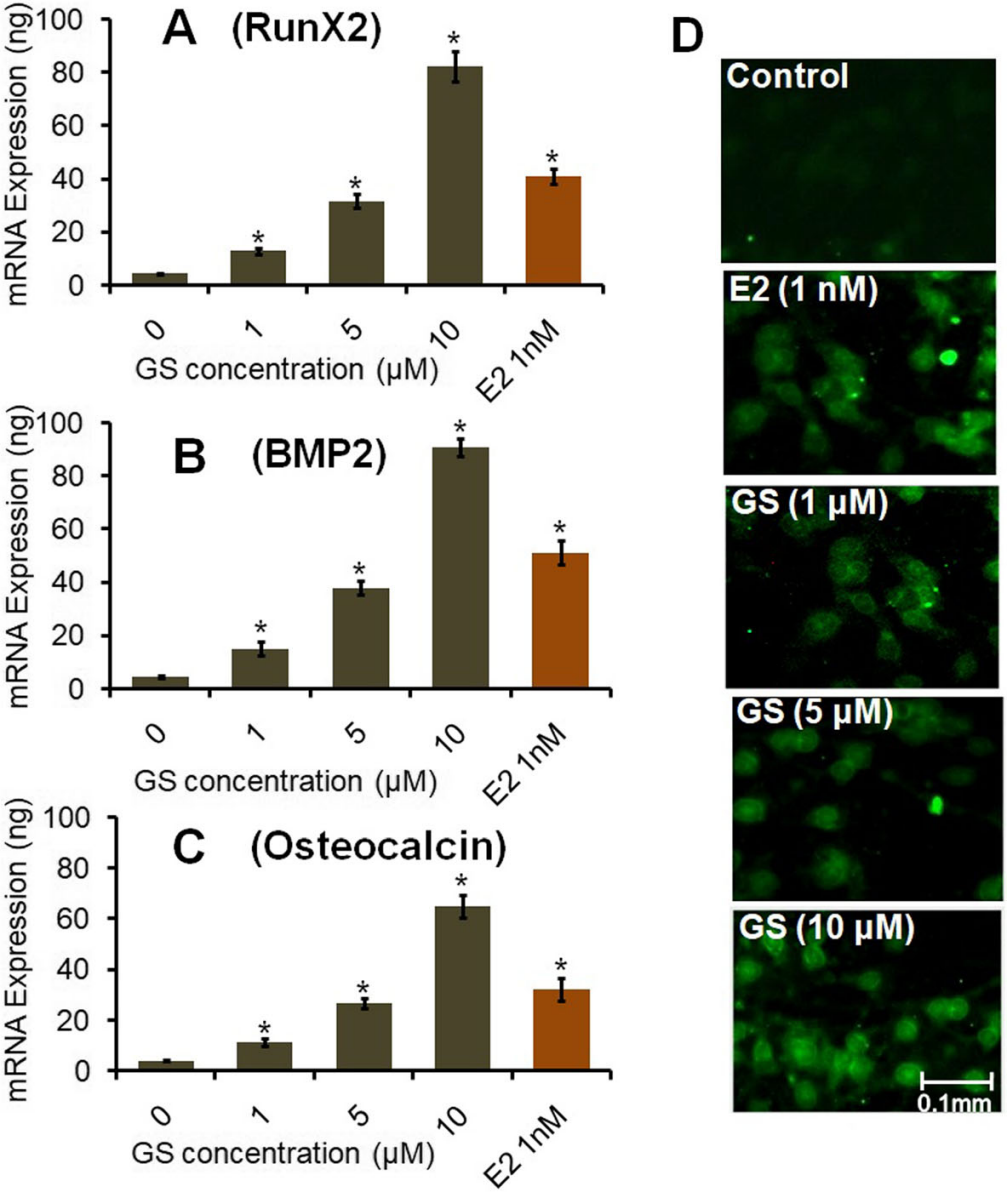
Effect of GS on mRNA levels of osteogenic genes and immunolocalization of the BMP2 protein in osteoblasts. Rat calvarial osteoblasts were treated with different concentrations 1, 5 and 10 μM of GS for 48 h. Absolute qPCR was performed to analyze the mRNA expressions of (**a**) Runx2 (**b**) BMP2 and (**c**) Osteocalcin genes in osteoblasts. **d** Photomicrographs show the immunofluorescence staining of BMP2 under inverted fluorescence microscope; magnification = 20x and scale bar = 0.1 mm. Values were obtained from three independent experiments performed in the triplicate set and expressed as mean ± SEM; ^*^P < 0.05

Osteogenic genes expression of bone cells treated with various concentrations of GS were observed by absolute qPCR at 48 h. As observed from the result, 1, 5 and 10 μM of GS elevated Runx2 expression significantly (*P <* 0.05) as compared to control (Fig. 5a). On the other hand, the mRNA level was found greater at 10 μM of GS treatment as compared to E2 treatment. Figure 5b shows that GS induces the expression of BMP2 significantly (*P <* 0.05) depending upon dose as compared to control. However, BMP2 expression at 10 μM of GS treatment was found to be greater as compared to 1 nM E2. This study also suggests that the osteogenic effect of GS may be mediated through increased production of BMP2. Furthermore, the downstream gene expression of Runx2 i.e. osteocalcin was also increased considerably in treatment groups compared to controls (Fig. 5c).

### Immunofluorescence staining of BMP2

The result of BMP2 immunofluorescence stain clearly indicated that GS elevated BMP2 level as a function of dose (Fig. 5d). This data is also compatible with qPCR data of BMP2 expression.

### Effect of GS on osteoclastogenesis

**Fig. 6:**
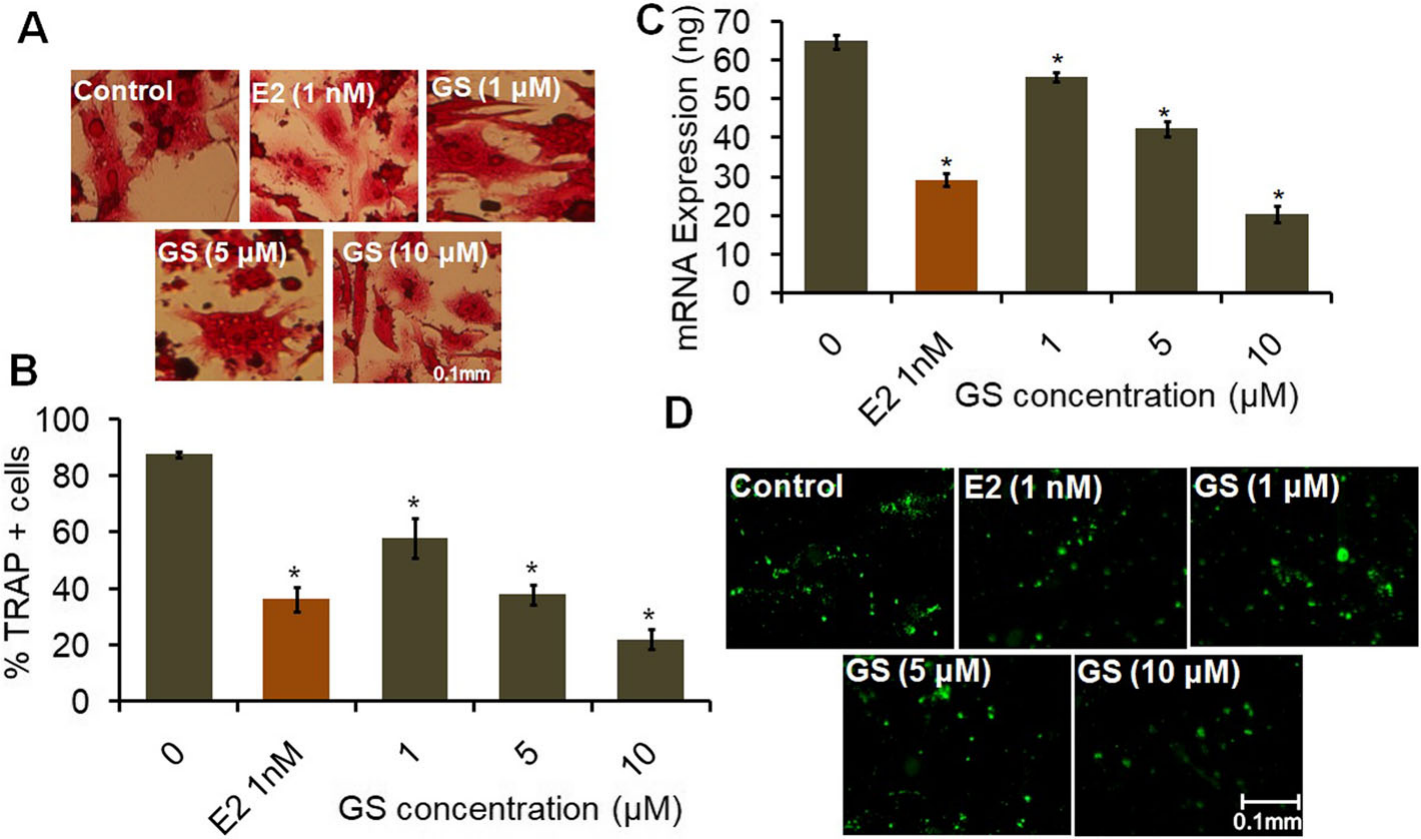
Inhibition of osteoclastogenesis in bone marrow progenitor cells and the effect of GS on cathepsin K expression of osteoclasts. **a** Bone marrow stem cells were treated at 1, 5 and 10 μM concentrations of GS for 9 days in differentiation medium for osteoclast formation. 1 nM of E2 was used as a positive control. Images were captured under a phase-contrast microscope. Scale bar = 0.1 mm (**b**) The number of TRAP-positive multinucleated cells were scored and represented as % TRAP-positive cells. Scale bar = 0.1 mm. **c** Absolute qPCR analysis for cathepsin K mRNAs expression (**d**) Immunofluorescence stain of cathepsin K. Values are expressed as mean ± SEM (*n* = 3); ^*^P < 0.05 compared with control

TRAP staining of osteoclasts exhibited that GS decreased the osteoclastogenesis by reducing the differentiation of multinucleated osteoclasts (Fig. 6a). As observed from quantitative results, GS reduced TRAP+ multinucleated cells number significantly depending upon dose when compared to control (Fig. 6b).

### Cathepsin K expression analysis

Osteoclast is characterized by the expression of cathepsin K which is a cysteine protease working as a potent collagenase. Results of expression analysis of cathepsin K indicated that GS decreased the cathepsin K expression dose-dependently compared to control (Fig. 6c). Although, 10 μM dose of GS reduced the level of cathepsin K significantly compared to E2 treatment. This data was consistent with result of TRAP stain. Furthermore, result of immunofluorescence stain of cathepsin K indicated that GS decreased the extent of cathepsin K stain dose-dependently compared to control (Fig. 6d). This result indicates that the flavonoid nature of GS may be responsible for the reduction of cathepsin K expression which may thus slow the degenerative changes of bone.

## Discussion

Bone remodeling is a tightly regulated process that is characterized by bone resorption by osteoclasts and bone formation by osteoblasts. Disruption of this finely regulated process leads to osteoporosis. Therefore, induction of bone formation and reduction of bone resorption would be the leading steps in osteoporosis therapy. In the present study, soy isoflavone genistein was used to its effect(s) on osteoblast proliferation and differentiation, oxidative stress and cell cycle progression in primary osteoblasts, as well as osteoclast attenuation.

Cell viability data revealed that GS treatment increased the growth of osteoblasts by increasing the growth and amount of live cells (Fig. 1a & b). The elongated and fusiform morphology of osteoblasts suggested the features of bone cell proliferation. This result is consistent with some previous reports where GS induced cell proliferation in primary mouse bone marrow-derived mesenchymal stem cells and human osteoblast cell line hFOB 1.19 [26, 27]. Alkaline phosphatase enzyme plays an essential role in the mineralization of osteoblasts and, thus, matrix mineralization is the vital process in bone formation. A previous study has investigated the combined effect of flavonoids daidzein and genistein increasing alkaline phosphatase activity in cultured MC3T3-E1 preosteoblastic cells [28]. This study is in agreement with the result of ALP activity of the present study and thus, suggests that GS has a stimulatory effect on osteoblasts differentiation. The mineralization promoting potential of GS observed in the present study has been found to be in agreement with another study which has reported that the dietary flavone baicalin causes a strong induction of alkaline phosphatase and increases mineralization in cultured rat osteoblasts [29].

Measurement of percent of cells in S phase or S-phase fraction determines whether cells are in a proliferative phase of cell cycle or not [30]. Cell cycle data displayed that GS caused accumulation of cells in S phase of cell cycle. Accumulation in S phase of cell cycle may have two meaning; either the cells grow or they are arrested because of DNA damage in apoptosis. Interestingly, the present data suggest that GS induces cell proliferation by accumulating the cells in S phase of cell cycle. These findings has been validated by other studies by examining induction of estrogen receptor expression and chondrocyte proliferation in S phase of the cell cycle [31, 32]. A recent study has reported that a biologically proliferative dose of *Cibotium barometz* polysaccharides stimulates in vitro chondrocyte proliferation via increasing the number of cells in S phase of cell cycle [33]. Thus, the present study furnishes satisfactory evidence that the accumulation of DNA in S phase may be a marker of cell proliferation.

Oxidative stress is characterized by high level of intracellular ROS production which disrupts the balance of the redox system [34]. Various studies have proved that ROS induces oxidative stress and plays a key modulator of bone cell function and regulator of bone pathophysiology [35, 36]. Isoflavone GS is known for its antioxidant activity in biological systems [37]. Interestingly, our data revealed that GS significantly inhibited the oxidant-induced overproduction of ROS in osteoblasts. In agreement with our results, a previous study has indicated that cordycepin, an active agent of *Cordyceps militaris,* displays antioxidant activity and prevents oxidative stress-induced inhibition of bone cell formation [38]. Similarly, an antioxidant fullerol has shown an osteogenesis promoting effect on human adipose-derived stem cells [39]. A recent study has reported the antioxidant effects of vitamin D3 under oxidative stress conditions which has been found to enhance the osteogenic differentiation of mesenchymal stem cells [40]. From these studies, it can be concluded that oxidative stress could be an important therapeutic target for bone cell remodeling and its associated function.

Osteogenesis is a well regulated process of osteoblasts and the factors generated by them could be used as indicators of various developmental stages. The most commonly used markers of osteoblast differentiation are alkaline phosphatase, Runx2, BMP2 and osteocalcin [41]. Quantitative real-time PCR analysis has shown that GS induces osteoblast differentiation by increasing the expression level of Runx2, BMP2 and osteocalcin genes (Fig. 5). Flavonoids are structurally very similar to estrogen and, therefore, they display strong estrogen-like activities. Hence, soy isoflavone genistein can be utilized as a substituent of estrogen. Because of its estrogen- like nature, GS may upregulate expression of ALP and collagen genes, and consequently, the expression level of Runx2 gene in osteoblasts. Runx2 is a non-collagenous key transcription factor regulating matrix mineralization of osteoblasts. Likewise, BMP2 gene regulates mineralization of osteoblasts and bone-specific alkaline phosphatase. Interestingly, a recent study has shown that active ingredients such as flavonoids, glycosides, terpenoids and phenols display estrogen-like biological activities, specifically affecting bone formation by regulating bone-specific matrix proteins (OCN, OPN, ALP, COL1), various transcription factors (Runx2, Osx, Cbfa1) and signaling pathways (BMP, MAPK) of osteoblasts [42]. Moreover, this study also shows that GS decreases osteoclastogenesis by reducing osteoclasts differentiation (Fig. 6). A previous study has shown that GS inhibits osteoclast differentiation by inhibiting the expression of TRAP, RANK, and calcitonin receptor in RAW 264.7 cell line [43]. Another study has found that aldehydic components of cinnamon bark extract strongly inhibits osteoclastic activity due to its estrogenic nature [44]. A recent study has also shown that flavonol herbacetin prevents formation and differentiation of osteoclasts with a decrease in the expression of RANK, TRAP, and cathepsin K in murine macrophage RAW264.7 cell line and bone marrow-derived macrophage cells [45]. These results have been found to be in agreement with the findings of the present study. Hence, the present study confirms the inhibitory mechanism of GS on osteoclast differentiation. These findings suggest that intake of GS isoflavone may be useful in bone health maintenance through osteoblastogenesis and osteoclast modulation.

## Conclusions

The present study highlighted the osteogenic efficacy of GS *via* its antioxidant effect and S-phase elevation in primary osteoblasts along with anti-osteoclastogenic effect against bone-marrow-derived osteoclasts. This study makes a significant contribution in unraveling the novel role(s) of GS in imparting protective effects against oxidative stress-induced bone loss and particularly, in the regulation of bone remodeling. Further studies are needed to validate GS as a potential therapeutic candidate and regenerative medicine for the treatment of age-related osteoporosis in humans.

## Supplementary information

**Additional file 1.**

## Data Availability

The datasets used and analyzed in the current study are included in this article.

## Abbreviations

GS: Genistein
SD: Sprague dawley
M-CSF: Macrophage colony stimulating factor
RANKL: Receptor activator of nuclear factor kappa-Β ligand
TRAP: Tartrate-resistant acid phosphatase
DCFH-DA: Dichloro-dihydro-fluorescein diacetate
Runx2: Runt-related transcription factor 2
BMP2: bone morphogenetic proteins 2
FBS: Fetal bovine serum
α-MEM: Alpha modification minimum essential medium
ALP: Alkaline phosphatase
pNPP: Para-nitro phenyl phosphate
qPCR: Quantitative polymerase chain reaction
BSA: Bovine serum albumin
