# Supplementary material for: Genistein contributes to cell cycle progression and regulates oxidative stress in primary culture of osteoblasts along with osteoclasts attenuation
Source: BMC Complement Med Ther. 2020 Sep 11;20:277. doi: 10.1186/s12906-020-03065-5 (PMC7488498; doi:10.1186/s12906-020-03065-5)
Supplement: Supplementary file 1 — Additional file 1. [file 12906_2020_3065_MOESM1_ESM.docx]

**Table S1: The sequence of gene primers of osteoblasts and osteoclasts**

| Target gene | Primer sequence |
| --- | --- |
| Runx2 | CCACAGAGCTATTAAAGTGACAGTG(F) AACAAACTAGGTTTAGAGTCATCAAGC(R) |
| Osteocalcin | CCCAATTGTGACGAGCTAGC(F) AACAAACTAGGTTTAGAGTCATCAAGC(R) |
| BMP2 | GATGGGCTTATTGACCAACC(F) TGGAGATTCAAGTTCCCAAA(R) |
| Cathepsin K | CCCAGACTCCATCGACTATCG(F) CTGTACCCTCTGCACTTAGCTGCC(R) |
